# Antiviral Activity of Bictegravir (GS-9883), a Novel Potent HIV-1 Integrase Strand Transfer Inhibitor with an Improved Resistance Profile

**DOI:** 10.1128/AAC.01474-16

**Published:** 2016-11-21

**Authors:** Manuel Tsiang, Gregg S. Jones, Joshua Goldsmith, Andrew Mulato, Derek Hansen, Elaine Kan, Luong Tsai, Rujuta A. Bam, George Stepan, Kirsten M. Stray, Anita Niedziela-Majka, Stephen R. Yant, Helen Yu, George Kukolj, Tomas Cihlar, Scott E. Lazerwith, Kirsten L. White, Haolun Jin

**Affiliations:** Gilead Sciences, Foster City, California, USA

## Abstract

Bictegravir (BIC; GS-9883), a novel, potent, once-daily, unboosted inhibitor of HIV-1 integrase (IN), specifically targets IN strand transfer activity (50% inhibitory concentration [IC_50_] of 7.5 ± 0.3 nM) and HIV-1 integration in cells. BIC exhibits potent and selective *in vitro* antiretroviral activity in both T-cell lines and primary human T lymphocytes, with 50% effective concentrations ranging from 1.5 to 2.4 nM and selectivity indices up to 8,700 relative to cytotoxicity. BIC exhibits synergistic *in vitro* antiviral effects in pairwise combinations with tenofovir alafenamide, emtricitabine, or darunavir and maintains potent antiviral activity against HIV-1 variants resistant to other classes of antiretrovirals. BIC displayed an *in vitro* resistance profile that was markedly improved compared to the integrase strand transfer inhibitors (INSTIs) raltegravir (RAL) and elvitegravir (EVG), and comparable to that of dolutegravir (DTG), against nine INSTI-resistant site-directed HIV-1 mutants. BIC displayed statistically improved antiviral activity relative to EVG, RAL, and DTG against a panel of 47 patient-derived HIV-1 isolates with high-level INSTI resistance; 13 of 47 tested isolates exhibited >2-fold lower resistance to BIC than DTG. In dose-escalation experiments conducted *in vitro*, BIC and DTG exhibited higher barriers to resistance than EVG, selecting for HIV-1 variants with reduced phenotypic susceptibility at days 71, 87, and 20, respectively. A recombinant virus with the BIC-selected M50I/R263K dual mutations in IN exhibited only 2.8-fold reduced susceptibility to BIC compared to wild-type virus. All BIC-selected variants exhibited low to intermediate levels of cross-resistance to RAL, DTG, and EVG (<8-fold) but remained susceptible to other classes of antiretrovirals. A high barrier to *in vitro* resistance emergence for both BIC and DTG was also observed in viral breakthrough studies in the presence of constant clinically relevant drug concentrations. The overall virologic profile of BIC supports its ongoing clinical investigation in combination with other antiretroviral agents for both treatment-naive and -experienced HIV-infected patients.

## INTRODUCTION

Integrase strand transfer inhibitors (INSTIs) are the latest class of antiretroviral drugs approved for the treatment of HIV-1 infection, and they inhibit HIV-1 replication by blocking the strand transfer step of viral DNA integration into the host genome (1–3). The first two INSTIs, raltegravir (RAL) and elvitegravir (EVG), have been approved for clinical use as components of combination antiretroviral therapy. Although both INSTIs have displayed good antiviral efficacy as components of combination regimens in the treatment of HIV-1 infections in randomized trials (3), RAL is dosed twice daily (4–6) while EVG is dosed once daily but requires coadministration with a pharmacokinetic enhancer to increase EVG systemic exposure (7–9). In addition, RAL and EVG have an overlapping resistance profile such that many viruses resistant to one drug are cross-resistant to the other drug, which ultimately precludes the sequential use of these two INSTIs (10–15).

Dolutegravir (DTG) was subsequently approved for treatment of HIV-1 in 2013. It is an unboosted INSTI with a higher barrier to resistance development and improved resistance profile relative to RAL and EVG and is dosed once daily for many patients (16–19). DTG is effective in naive patients and did not induce resistance development in registrational studies of naive and suppressed switch patients (20–24), but it did show emergence of additional INSTI resistance in a trial of patients with EVG and RAL resistance (24) and in recent case reports of INSTI resistance in treatment-naïve and treatment-experienced, but INSTI-naïve, patients (25). In addition, DTG must be dosed twice daily when coadministered with cytochrome P450 (CYP) and/or UDP glucuronosyltransferase (UGT) inducers (e.g., efavirenz [EFV], fosamprenavir/ritonavir, tipranavir/ritonavir, or rifampin) and in patients with documented or suspected INSTI-associated resistance (24, 26). In addition, DTG increases human serum creatinine levels by about 10% via inhibition of organic cation transporter 2 (OCT2; 50% inhibitory dose [IC_50_] of 1.93 μM) in the proximal tubule of the kidney (27, 28). Consequently, novel INSTIs with improved pharmacokinetics (including minimized drug-drug interactions and effects on drug transporters), once-daily dosing, improved tolerability, high efficacy against INSTI-associated resistance, and smaller pill size would be useful in the treatment of HIV.

Bictegravir (BIC; GS-9883) is a novel INSTI that has recently advanced into registrational clinical trials in combination with tenofovir alafenamide (TAF) and emtricitabine (FTC) in a single-tablet formulation for the treatment of HIV-1 infection. In this report, we describe the *in vitro* biological characterization of BIC and show its potent activity against laboratory strains and clinical isolates of HIV-1, a higher barrier to resistance development than RAL and EVG, and a statistically improved resistance profile compared to those of RAL, EVG, and DTG against a set of patient-derived INSTI-resistant viral isolates.

## MATERIALS AND METHODS

### Compounds.

BIC, DTG, RAL, EVG, darunavir (DRV), atazanavir (ATV), tenofovir (TFV), TAF, FTC, rilpivirine (RPV), 2′-C-methyladenosine (2′-CMeA), 2′-fluoro-2′-deoxyguanosine (2′-FDG), rupintrivir, and YM-53403 were synthesized at Gilead Sciences, Inc. Ribavirin (RBV) (catalog number R-9644), stavudine (d4T) (catalog number D-1413), and zidovudine (AZT) (catalog number A-2169) were purchased from Sigma (St. Louis, MO). EFV was purchased from Toronto Research Chemicals Inc. (catalog number E425000; North York, Ontario, Canada).

### Cells.

MT-2 cells were obtained from Stanford University, and MT-4 cells were obtained from the NIH AIDS Research and Reference Reagent Program (Germantown, MD). MT-2 and MT-4 cells were maintained in RPMI 1640 medium supplemented with 10% heat-inactivated fetal bovine serum (FBS) and antibiotics. The SODk1 2G cell line that produces vesicular stomatitis virus glycoprotein (VSV-G)-pseudotyped viral particles used in single-cycle infection was licensed from the Salk Institute, La Jolla, CA (29, 30). SODk1 2G cells were maintained in Dulbecco's modified Eagle's medium (DMEM) supplemented with 10% tetracycline-free fetal bovine serum (FBS) (Clontech, Mountain View, CA), 1 mM glutamine, 1 mM pyruvate, 1 μg/ml doxycycline, and antibiotics. Production of the pseudotyped particles from SODk1 2G cells has been previously described (29).

Freshly isolated human peripheral blood mononuclear cells (PBMCs) were obtained from healthy volunteers (supplied by AllCells, Inc., Alameda, CA). Blood donors were negative for HIV-1, hepatitis B, and hepatitis C viral infections. CD4^+^ T lymphocytes were isolated by negative selection using a magnetically labeled antibody cocktail (STEMCELL Technologies Inc., Vancouver, Canada) and cultured in RPMI cell culture media (Life Technologies, Grand Island, NY) supplemented with 10% FBS. CD4^+^ T cells were activated for 48 h at 37°C by addition of 1 μg/ml phytohemagglutinin (PHA; Sigma, St. Louis, MO) and 5 ng/ml interleukin-2 (IL-2; Roche Diagnostics, Indianapolis, IN). Monocytes were isolated by negative selection using a magnetically labeled antibody cocktail (STEMCELL Technologies Inc., Vancouver, Canada), seeded into 96-well plates at a density of 2 × 10^5^ cells per well, and then incubated for 8 days at 37°C in RPMI cell culture medium supplemented with 10% FBS, 5% human serum (Sigma, St. Louis, MO), and 50 ng/ml granulocyte-macrophage colony-stimulating factor (GM-CSF; R&D Systems, Minneapolis, MN) to permit their differentiation into macrophages. Human PBMCs were used for cytotoxicity assays after culturing for 48 h in either complete RPMI medium alone (resting conditions) or RPMI containing 1 μg/ml PHA and 5 ng/ml IL-2 (activated conditions).

### HIV strains.

HIV-1 strain IIIb was obtained from Advanced Biotechnologies Inc. (Columbia, MD). HIV-1 BaL virus (Advanced Biotechnologies, Columbia, MD) was passaged in human PBMCs. HIV-1 recombinant strains carrying mutations were prepared by transfecting infectious pLAI-based or HXB2-based cDNA clones into 293T cells, followed by virus amplification in MT-4 cells and harvesting of the cell supernatants. The nucleoside reverse transcriptase inhibitor (NRTI)-resistant viruses encoding reverse transcriptase (RT) mutation K65R were obtained from Mark Wainberg (McGill AIDS Center, Montreal, Canada). The NRTI-resistant viruses encoding RT mutation M184V were constructed by site-directed mutagenesis (31). The virus 6TAMs, containing six resistance mutations in HIV-1 RT (M41L, D67N, K70R, L210W, T215Y, and K219Q) that confer resistance to thymidine analogs, was constructed by cloning a PCR-amplified RT-encoding fragment from HIV-infected patient-derived plasma (31). The nonnucleoside reverse transcriptase inhibitor (NNRTI)-resistant viruses encoding RT mutation K103N, Y181C, Y188L, or L100I/Y181C were constructed by site-directed mutagenesis. The protease inhibitor (PI)-resistant mutant viruses encoding mutations in the HIV-1 protease-coding sequence, L10F/M46I/I50V, I84V/L90M, G48V/I54V/V82S, and G48V/V82A/L90M, were produced in electroporated SupT1 cells via homologous recombination (32). Mutations conferring resistance to INSTIs were introduced into the infectious wild-type HIV-1 DNA clone HXB2 by site-directed mutagenesis. Mutation(s) was confirmed by sequencing. The HIV strains used for phenotyping by Southern Research Institute, Frederick, MD, were the following (name and GenBank accession number): 92RW016 (AF009409), 92UG037 (AB253428), BaL (AY713409), 89BZ_167 (AY173956), 91US001 (AY173952), 91US004 (AY173955), 93IN905 (AF067158), 98US_MSC5016 (AY444801), 98UG_57128 (AF484502), 99UG_A07412M1 (AF484477), 96TH_M02138 (AY713424), 96TH_NI1046 (AY713421), 93BR020 (AF005494), 01CM1475MV (AY371138), and CDC 310319 (AY965902).

### Expression and purification of HIV-1 IN.

Recombinant IN containing an N-terminal 6His tag was expressed in BL21(DE3) pLysS bacteria. The details of expression and purification of HIV-1 IN were previously described (33). The purity of IN preparations was generally >95%.

### IN strand transfer and 3′-processing assays.

The strand transfer activity of IN was measured using an assay based on homogeneous time-resolved fluorescence resonance energy transfer (HTRF) (34) and has been previously described in detail (35). An HTRF-based 3′-processing assay was performed essentially as described for the IN strand transfer assay using a specially designed nonprocessed donor DNA in the absence of target DNA (35).

### Anti-HIV and cytotoxicity assays.

For the antiviral assay utilizing MT-2 and MT-4 cells, 50 μl of a 2× test concentration of 5-fold serially diluted compound in culture medium with 10% FBS was added to each well of a 96-well plate (9 concentrations) in triplicate. MT-2 and MT-4 cells were infected with HIV-1 IIIb at a multiplicity of infection (MOI) of 0.01 for 3 h. Fifty microliters of infected cell suspension in culture medium with 10% FBS (∼1.5 × 10^4^ cells) then was added to each well containing 50 μl of diluted compound. The plates were then incubated at 37°C for 5 days. After 5 days of incubation, 100 μl of CellTiter-Glo reagent (Promega Biosciences, Inc., Madison, WI) was added to each well containing MT-2 or MT-4 cells. Cell lysis was carried out by incubation at room temperature for 10 min and chemiluminescence was read. For compound cytotoxicity assessment, the protocol was identical except that uninfected cells were used and compounds were serially diluted 3-fold.

In order to accurately determine the Hill slope of the antiviral dose response, compounds were serially diluted in dimethyl sulfoxide (DMSO) with steps of 1.5-fold in 2 overlapping serial dilution series to generate a 40-point compound dose range. Each compound series was prepared several times independently in quadruplicate to capture the variations in Hill slope associated with preparation of compound serial dilutions. The assay was performed in a 384-well format. MT-4 cells were infected in bulk at the same MOI as that described above, and the plates were developed with CellTiter-Glo reagent 5 days postinfection.

For the antiviral assay, CD4^+^ T lymphocytes at a density of 4 × 10^6^ cells per ml were infected in bulk culture with HIV-1 BaL at an MOI of 15 ng p24 equivalent per million cells for 3 h at 37°C. Three hours after viral adsorption, the cells were washed once with the RPMI medium and seeded into 96-well plates at a density of 2 × 10^5^ cells per well. A 3-fold serial dilution of test compounds in RPMI medium was added to triplicate wells and incubated at 37°C for 5 days. Human monocytes were seeded on 96-well plates at a density of 3 × 10^5^ cells per well, differentiated into adherent macrophages, and then infected with HIV-1 BaL virus in 50 μl RPMI for 3 h at 37°C. Cells were washed once with complete RPMI medium and incubated for 12 days at 37°C with 200 μl RPMI medium containing 10% FBS, 50 ng/ml GM-CSF, and serially diluted compound. Macrophages received two changes of drug-containing medium during this 12-day incubation period. The supernatants derived from the CD4^+^ T-cell and macrophage cultures were harvested 6 and 12 days postinfection, respectively, and the amount of HIV present was quantified by p24 enzyme-linked immunosorbent assay (ELISA) (PerkinElmer, Waltham, MA).

Antiviral assay in fresh human PBMCs with RT endpoint was performed by Southern Research Institute, Frederick, MD, as a contracted research study.

### Assay for 2-LTR circles, late reverse transcription products, and integration junctions.

Quantification of 2-long terminal repeat (LTR) circles, late reverse transcription products, and integration junctions was adapted from a previously described method (30). MT-2 cells were infected in bulk culture with HIV-1 IIIb at an MOI of 10 in the presence of 10 μg/ml Polybrene (Santa Cruz Biotechnology, Santa Cruz, CA) and at a cell density of 2 × 10^6^ cells/ml for 3 h at 37°C. Three hours after viral adsorption, the infected cells were washed twice with 20 ml of RPMI 1640 medium containing 10% FBS and antibiotics and seeded in 6-well plates in 2 ml of culture medium at 1 × 10^6^ cells per well. Infected MT-2 cells received either DMSO (mock-treated control) or one of four compounds (BIC, DTG, EFV, or DRV) at a final concentration greater than or equal to 20 times their respective antiviral 50% effective concentration (EC_50_). These plates were incubated at 37°C for either 12 h (for late reverse transcription product quantification) or 24 h (for 2-LTR circle and Alu-LTR product quantification), after which time the cells were harvested for total DNA isolation. DNA was extracted from each well using the QIAamp DNA minikit (Qiagen, Valencia, CA) and collected as a 100-μl eluate. TaqMan real-time PCR-quantified 2-LTR junctions (2-LTR circles), late reverse transcription products, and integration junctions (Alu-LTR) were normalized to the level of host globin gene in each sample. Quantification by PCR was performed on the ViiA 7 real-time PCR system (Life Technologies, Grand Island, NY).

### Phenotypic analysis of clinical isolates.

Forty-seven resistance test vectors containing patient-derived IN coding regions were constructed using standard Monogram Biosciences protocols. Phenotypic data for BIC, DTG, EVG, and RAL (EC_50_s) in these clinical isolates were obtained through a contract study by Monogram Biosciences (South San Francisco, CA) using the PhenoSense IN assay. The fold change from the NL4-3 wild-type reference vector values was determined. Statistical significance was determined using the Student two-tailed *t* test.

### HIV-1 resistance selections.

Resistance selections were performed in 6-well tissue culture plates. MT-2 cells were seeded at a density of 0.5 × 10^6^ cells per well in 5 ml of culture medium. Compounds were added at final concentrations corresponding to their antiviral EC_50_ or twice the EC_50_. HIV-1 IIIb was used at an MOI of ∼0.01. The cultures were incubated at 37°C and split 1:2 to 1:3 once or twice a week depending on the growth status of the cells. The cytopathic effect (CPE) manifested as syncytium formation was used to monitor progression of infections. Virus was harvested and transferred to fresh MT-2 cells in the presence of the same compound but at a 2-fold higher concentration. Successive viral passages were obtained by repeating this procedure. The duration of each passage ranged from 10 to 15 days.

### Genotypic analysis of inhibitor-selected HIV-1 variants.

Genotypic changes under selective pressure were assessed by population sequencing of the entire IN coding region of viruses at different stages of the selection process. Total RNA isolated from virus supernatant collected from various passages was used for the amplification by RT-PCR of a 998-bp fragment encoding HIV-1 IN. This fragment spans nucleotide 1600 of the reverse transcriptase coding region to nucleotide 107 of the *vif* gene and includes the entire IN coding region. Total RNA was extracted from 140 μl of virus supernatant using the QIAamp viral RNA minikit (Qiagen, Valencia, CA) and eluted in 60 μl of AVE buffer. The RT-PCR was performed according to the manufacturer's protocol using a Titan One tube RT-PCR kit (Roche Applied Science, Indianapolis, IN). The two primers used to amplify the cDNA have the following sequences: p041, 5′-GCATGGGTACCAGCACACAAAG-3′; and p042, 5′-CTAGCTTTCCCTGAAACATACATATGGTG-3′. The RT-PCR product was purified using a QIAquick PCR purification kit (Qiagen, Valencia, CA), and sequencing was performed by Elim Biopharmaceuticals, Inc. (Hayward, CA), using primers p041, p042, INTseq3 (5′-GCAGGAAGATGGCCAG-3′), and INTseq5Rev (5′-GAGGAGCTTTGCTGGTCC-3′).

### HIV breakthrough selections in MT-2 cells.

MT-2 cells were infected in bulk culture with HIV-1 IIIb at an MOI of 0.05 and at a density of 2 ×10^6^ cells/ml for 2 h at 37°C. Two hours after virus adsorption, the cells were centrifuged for 5 min at 2,000 rpm, resuspended in fresh RPMI cell culture medium (containing 10% FBS), and seeded into 24-well plates at a cell density of 2 × 10^5^ cells per well. Test compounds were diluted in RPMI medium and added to quadruplicate wells to achieve a final volume of 1 ml/well. The final (1×) drug concentrations assessed were equal to 2.5× and 5× EC_95_ for both DTG (32 nM and 16 nM, respectively) and BIC (42 nM and 21 nM, respectively), 4× EC_95_ for FTC (25 μM), and 5× EC_95_ for EVG (48 nM). Viral breakthrough was assessed over a period of 32 days, during which cell cultures were maintained by routine passaging with a 1 to 5 split every 3 to 4 days by the addition of 800 μl fresh drug-containing RPMI medium. At each time point during the course of the assay (i.e., days 4, 7, 11, 14, 18, 21, 25, 28, and 32 postinfection), wells were visually inspected under high magnification for the development of virus-induced CPE. For wells in which >80% of the cell culture showed evidence of CPE, the viral supernatant was harvested and the viral RNA products purified using the QIAamp viral RNA minikit (Qiagen, Redwood City, CA). The IN coding region in each sample was then amplified using the Qiagen OneStep RT-PCR kit and subjected to DNA sequencing (ELIM Biopharmaceuticals, Inc., Hayward, CA) to identify any potential resistance-associated mutations.

## RESULTS

### BIC inhibits HIV-1 IN.

The structures of the approved INSTIs BIC, EVG, and DTG are shown in Table 1 (1). Bictegravir differs from previously known structures in that it contains a unique bridged bicyclic ring and a distinct benzyl tail consisting of a trisubstituted 2,4,6-trifluorobenzyl moiety. These changes resulted in reduced pregnane X receptor (PXR) activation, minimizing the risk for drug-drug interactions with other coadministered agents, and increased plasma protein binding. The increase in protein binding contributed to lower *in vivo* clearance observed in preclinical as well as in clinical studies (36). The changes giving rise to BIC were also found to improve solubility (which is important for high oral drug absorption, especially at increased doses) and antiviral activity against many known INSTI-resistant viruses.

**TABLE 1:**
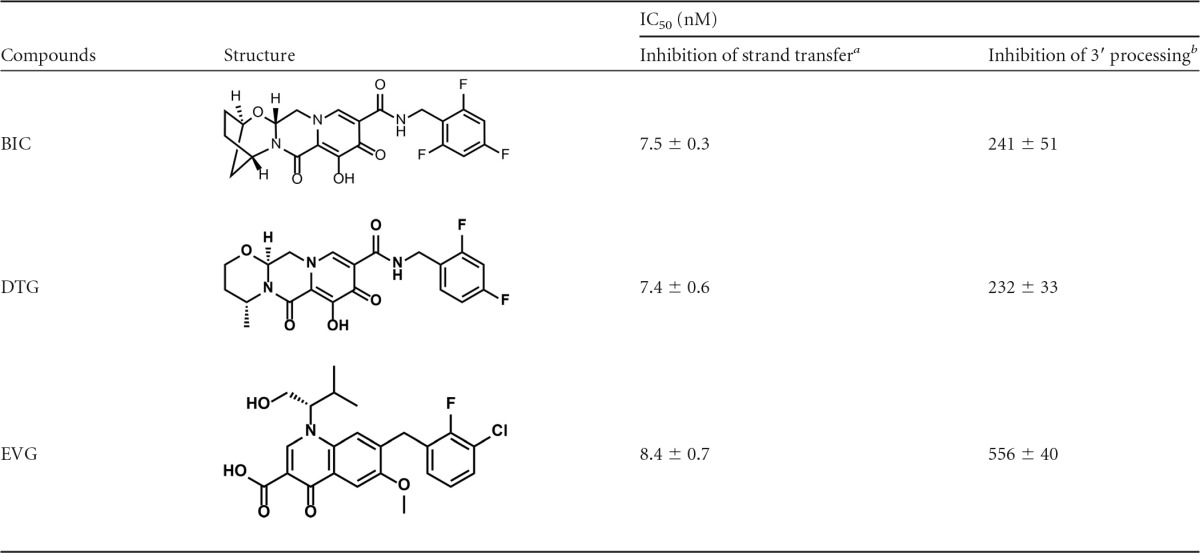
Inhibition of HIV-1 integrase enzymatic activities

^a^ The data represent the means ± SD from 3 independent experiments performed in triplicate.

^b^ The data represent the means ± SD from 5 independent experiments performed in triplicate.

The inhibitory activity of BIC was evaluated in biochemical assays that measured the 3′ processing and strand transfer activities of purified recombinant HIV-1 IN (Table 1). BIC inhibited the strand transfer activity with an IC_50_ of 7.5 ± 0.3 nM, comparable to those of EVG and DTG (16, 37). Relative to its inhibition of strand transfer activity, BIC was a much weaker inhibitor of 3′-processing activity of HIV-1 IN, with an IC_50_ of 241 ± 51 nM.

The end product of authentic HIV-1 DNA integration into host cell chromosomes can be assessed by PCR amplification of the junction between proviral DNA and the site of integration using the Alu-LTR primers (30). Furthermore, a distinct by-product of HIV DNA integration failure is characterized by the formation of circular HIV DNA in which the 2-LTRs are juxtaposed (30). BIC inhibited HIV-1 DNA integration in cell culture as measured by both of these parameters. BIC enhanced the accumulation of 2-LTR circles ∼5-fold relative to the mock-treated control and reduced the amount of authentic integration products in infected cells by 100-fold (Table 2). Although BIC profoundly decreased integration within host cell DNA, it did not affect viral DNA synthesis as measured by the accumulation of late reverse transcription products, which further supports its mechanism of action of selective and targeted inhibition of HIV-1 integration (Table 2). DTG, an INSTI comparator control, also decreased integration products and increased 2-LTR circles, whereas EFV, an NNRTI, decreased the late reverse transcription products with subsequent downstream effects on both 2-LTR circles and integration products. DRV, a protease inhibitor, had no effect on either DNA synthesis or integration.

**TABLE 2:** Inhibition of HIV-1 integration in MT-2 cells by BIC

Compound^*a*^	Integration failure of 2-LTR circles^*b*^ (fold change)	Assessment of direct effect on integration^*b*^ (fold change)	
		Late reverse transcription products	Alu-LTR integration junctions
DMSO	1.00 ± 0.42	1.00 ± 0.19	1.00 ± 0.41
BIC	5.55 ± 0.34*	0.81 ± 0.09	0.01 ± 0.01*
DTG	2.47 ± 1.26*	0.86 ± 0.08	0.01 ± 0.01*
EFV	0.05 ± 0.04*	0.21 ± 0.07*	0.10 ± 0.05*
DRV	0.57 ± 0.24	0.96 ± 0.11	0.72 ± 0.31

a Compounds were applied to HIV-1 (IIIb)-infected MT-2 cultures at >20-fold of their respective antiviral EC_50_. Means and SD were determined from ≥3 experiments.

b The quantitative PCR data of HIV target sequences were normalized against the corresponding quantitative PCR data for the globin gene from the same sample and represent the means from two independent experiments. Fold change is relative to the DMSO control. *, *P* < 0.05.

### BIC exhibits potent and selective anti-HIV activity and low cytotoxicity.

The ability of BIC to inhibit HIV-1 replication *in vitro* was evaluated in lymphoblastoid T-cell lines MT-2 and MT-4 and in primary human T lymphocytes and macrophages (Tables 3 and 4). BIC potently inhibited HIV-1 replication in both MT-2 and MT-4 cells with EC_50_s of 1.5 and 2.4 nM, respectively, and selectivity indices (50% cytotoxic concentration [CC_50_]/EC_50_) of ∼6,800 in MT-2 cells and ∼1,500 in MT-4 cells (Table 3). In both cell lines, BIC showed antiretroviral activity similar to that of DTG. Independently, the EC_95_ value for the inhibition of HIV-1 replication was calculated using a Hill slope determined from the curve fit to a high-density antiviral dose response in MT-4 cells using high-density serial dilutions of test compounds (Table 3). BIC inhibition in MT-4 cells showed a Hill slope of ∼2.1, yielding an EC_95_ value of 8.3 nM. Parallel profiling of DTG resulted in a comparable EC_95_ value of 7.4 nM. The EC_95_ for BIC calculated from the high-density antiviral dose response was used in conjunction with the human serum shift determined by equilibrium dialysis (EQDS) to obtain a protein binding-adjusted EC_95_ (PAEC_95_) value of 361 nM. The PAEC_95_ value was subsequently used for the estimation of clinical inhibitory quotient (IQ) to guide the selection of human dose.

**TABLE 3:** Antiviral activity and selectivity of bictegravir in MT-2 and MT-4 cell lines

Compound	MT-2 cells^*a*^			MT-4 cells^*a*^			MT-4 cells^*b*^				
	EC_50_ (nM)	CC_50_ (nM)	Selectivity	EC_50_ (nM)	CC_50_ (nM)	Selectivity	EC_50_ (nM)	Hill slope	EC_95_ (nM)	EQDS shift^*c*^ (fold change)	PAEC_95_ (nM)
BIC	1.5 ± 0.2	10,300 ± 2,500	6,867	2.4 ± 0.4	3,659 ± 93	1,525	1.9 ± 0.6	2.1 ± 0.3	8.3	43.6 ± 7.7	361
DTG	1.5 ± 0.2	5,350 ± 200	3,567	1.5 ± 0.3	14,677 ± 1,899	9,785	1.7 ± 0.2	2.0 ± 0.3	7.4	27.5 ± 5.8	204

a EC_50_ and CC_50_ values represent the means ± SD from at least 4 independent determinations in triplicate. Test compounds were 3-fold serially diluted.

b EC_50_ and Hill slope values represent the means ± SD from 8 independent determinations in quadruplicate. Test compounds were 1.24-fold serially diluted.

c Values represent means ± SD from 3 independent determinations.

**TABLE 4:** Antiviral activity and selectivity of bictegravir in human primary T cells and macrophages

Compound	CD4^+^ T lymphocytes^*a*^			Macrophages^*a*^			CC_50_^*b*^ (nM)	
	EC_50_ (nM)	CC_50_ (nM)	Selectivity	EC_50_ (nM)	CC_50_ (nM)	Selectivity	Resting PBMCs	Activated PBMCs^*c*^
BIC	1.5 ± 0.3	13,000 ± 4,000	8,700	6.6 ± 4.1	29,800 ± 7,700	4,500	8,400 ± 1,900	5,700 ± 2,200
DTG	1.0 ± 0.3	52,000 ± 8,500	52,000	3.1 ± 2.5	24,900 ± 1,200	8,000	30,600 ± 10,000	23,900 ± 3,100

a EC_50_ and CC_50_ values represent the means ± SD from triplicate measurements in four independent donors.

b CC_50_ values represent the means ± SD from triplicate measurements in three independent donors over 6 days.

c Activated for 2 days with IL-2/PHA prior to drug treatment.

BIC exhibits potent antiviral effects in both primary CD4^+^ T lymphocytes and monocyte-derived macrophages, with EC_50_s of 1.5 ± 0.3 nM and 6.6 ± 4.1 nM, respectively, which are comparable to values obtained in T-cell lines (Table 4). BIC also displayed high potency against 14 clinical isolates of HIV-1 and one isolate of HIV-2 in freshly isolated human PBMCs, with a mean EC_50_ of 0.81 nM and a range of EC_50_s between 0.04 and 1.7 nM (Table 5). In contrast, in cell-based assays, BIC had no antiviral activity against nonretroviruses, such as hepatitis B and C viruses (see Table S1 in the supplemental material), and respiratory viruses (see Table S2), including influenza A and B viruses, human rhinovirus, and respiratory syncytial virus.

**TABLE 5:** Antiviral activity of bictegravir against HIV clinical isolates

Virus	Isolate	EC_50_^*a*^ (nM)		
		BIC	DTG	AZT
HIV-1 subtype				
A	92RW016	0.71 ± 0.26	0.34 ± 0.09	2.0 ± 0.3
A	92UG037	1.5 ± 0.6	0.66 ± 0.05	11.2 ± 3.7
B	BaL	0.35 ± 0.12	0.32 ± 0.11	3.8 ± 0.6
B	89BZ_167	1.0 ± 0.5	1.4 ± 0.4	3.6 ± 0.8
B	91US001	0.89 ± 0.17	0.80 ± 0.48	5.5 ± 2.5
B	91US004	1.2 ± 0.4	1.0 ± 0.4	2.6 ± 1.5
C	93IN905	0.15 ± 0.01	0.17 ± 0.03	1.6 ± 0.7
C	98US_MSC5016	1.2 ± 0.2	0.75 ± 0.09	7.9 ± 4.4
D	98UG_57128	0.33 ± 0.01	0.32 ± 0.48	0.07 ± 0.03
D	99UG_A07412M1	1.1 ± 0.1	0.99 ± 0.56	9.9 ± 1.3
E	96TH_M02138	0.51 ± 0.17	0.11 ± 0.01	5.5 ± 0.4
E	96TH_NI1046	0.25 ± 0.06	0.25 ± 0.08	2.3 ± 0.7
F	93BR020	1.2 ± 0.5	0.40 ± 0.19	8.1 ± 7.1
G	01CM1475MV	0.04 ± 0.03	0.09 ± 0.04	1.2 ± 0.6
HIV-2	CDC 310319	1.7 ± 1.0	2.5 ± 0.5	9.3 ± 4.6

a EC_50_s represent the means and standard deviations from triplicate measurements in human PBMCs.

The cytotoxicity of BIC measured in primary CD4^+^ T cells and monocyte-derived macrophages translated into selectivity indices (CC_50_/EC_50_) of ∼8,700-fold for CD4^+^ T lymphocytes and of ∼4,500-fold for macrophages (Table 4). The cytotoxicity of BIC was also assessed with fresh human PBMCs in the resting state and upon mitogen activation. The cytotoxicity of BIC in resting PBMCs (CC_50_ of 8.4 μM) was similar to that observed in primary CD4^+^ T cells and macrophages and did not significantly change upon the mitogenic activation of PBMCs (CC_50_ of 5.7 μM) (Table 4). BIC is also devoid of cytotoxicity in nontarget cell lines such as hepatoma cell lines (Huh7 and HepG2), a prostate carcinoma cell line (PC3), a normal embryonic lung fibroblast line (MRC5), and primary human hepatocytes (see Table S3 in the supplemental material).

### BIC is synergistic in combination with approved HIV-1 antiviral drugs.

To assess the antiviral activity of BIC in combination with clinically approved agents from other antiretroviral classes, the compound was tested in pairwise combinations with a panel of selected drugs, including NRTIs, PIs, and INSTIs. Specifically, the antiviral activity of BIC was evaluated in combination with five antiretroviral drugs, including TAF, FTC, DRV, RAL, and EVG, in a 5-day cytopathic assay using MT-2 cells acutely infected with HIV-1. The effect of combining any two drugs was analyzed by the MacSynergy II software (38, 39). EVG-TAF, ribavirin-stavudine, and BIC-BIC combinations were used as synergistic, antagonistic, and additive controls, respectively.

The combinations of BIC with NRTIs (TAF and FTC) or with PIs (DRV) were highly synergistic (see Table S4 in the supplemental material). When combined with other INSTIs (RAL and EVG), BIC showed additive anti-HIV activity.

### BIC is active against INSTI-resistant mutants of HIV-1.

Nine prospectively defined mutant viruses that represent the major RAL- and EVG-resistant escape variants (13, 14, 40), containing both single and double mutants in the IN, were used to evaluate candidate compounds during the discovery efforts to identify BIC. The set of tested mutations included E92Q, Y143R, Q148R, N155H, R263K, E138K/Q148K, G140S/Q148R, E92Q/N155H, and Q148R/N155H. R263K was included because it was selected *in vitro* with DTG (41) and has been reported to emerge in two patient cases following treatment with DTG (50 mg once a day) in combination with a background ARV regimen (42). All tested mutant variants displayed less resistance against DTG than either RAL or EVG (Table 6). In a side-by-side comparison with DTG, the same variants displayed comparable or, for the G140S/Q148R mutant, even lower levels of resistance against BIC (Table 6). BIC maintained potent antiviral activity against all of the single IN mutants tested. Among the double mutants, all exhibited similar or slightly lower levels of resistance to BIC (1.3- to 9-fold) compared to DTG (2.1- to 10-fold). Collectively, these data indicate that BIC has the potential to be clinically active against the majority of known HIV-1 variants with INSTI-associated mutations. The NNRTI EFV was used as a control that remained active against all tested INSTI-resistant mutants.

**TABLE 6:**
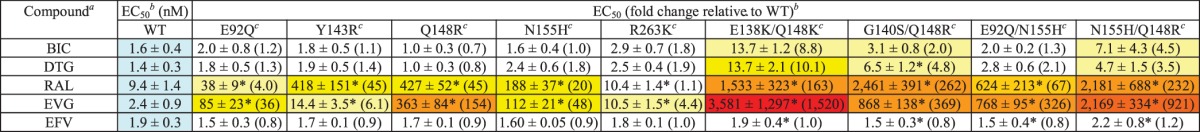
Activity of bictegravir against INSTI-resistant HIV-1 mutants

^a^ An asterisk indicates a *P* value of <0.05 versus BIC.

^b^ EC_50_ (±SD) and fold change represent the means from at least 3 independent determinations in triplicate. Wild-type virus was generated from HIV-1 DNA clone HXB2. The color shadings subdivide the fold change of resistance into six levels: light blue, wild type; light yellow, >2-fold; bright yellow, >10-fold; light orange, >50-fold; orange, >100-fold; red, >500-fold.

^c^ HIV-1 integrase (IN) mutant.

### BIC exhibits an improved profile relative to DTG against patient-derived isolates resistant to INSTIs.

**FIG 1:**
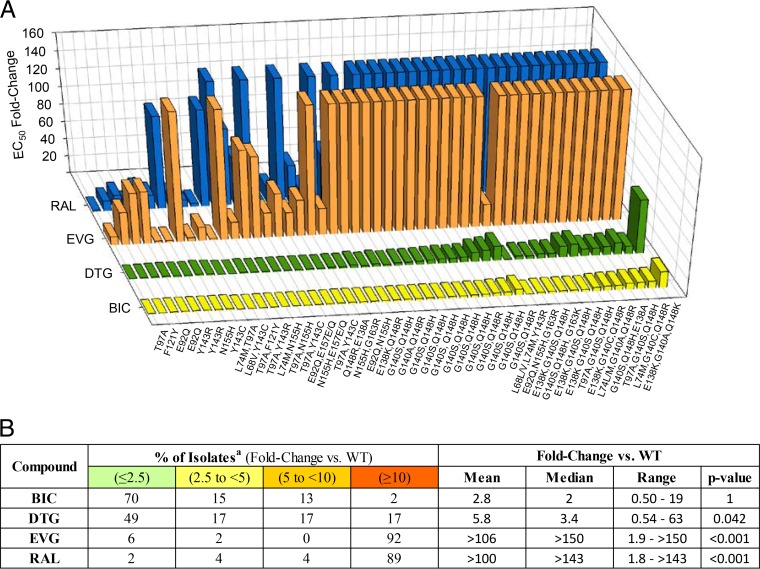
Resistance profile of BIC and other INSTIs against 47 HIV-1 patient-derived isolates with INSTI resistance mutations. (A) Bar graph of fold change in resistance. (B) Stratification of the clinical isolates based on fold change in resistance. Primary and other INSTI resistance mutations are listed. Primary INSTI resistance mutations are T66I/A/K, E92Q/G, T97A, Y143C/H/R, S147G, Q148H/K/R, and N155H, and other INSTI resistance mutations are H51Y, L68I/V, V72A/N/T, L74M, Q95K/R, F121C/Y, A128T, E138A/K, G140A/C/S, P145S, Q146I/K/L/P/R, V151L/A, S153A/F/Y, E157K/Q, G163K/R, E170A, and R263K in IN. Susceptibility was determined as the fold change in EC_50_ versus that of the NL4-3 wild-type vector by Monogram Biosciences, Inc. The biological or lower clinical cutoffs for reduced susceptibility in this assay are 4.0 for DTG, 1.5 for RAL, and 2.5 for EVG. No cutoff has been determined for BIC.

To further characterize its resistance profile, BIC potency was assessed against 47 HIV-1 polyclonal variants derived from clinical isolates (representing all available INSTI resistance variants in the library) with phenotypic resistance to INSTIs (Fig. 1; see also Table S5 in the supplemental material). Based on data from these patient-derived isolates, BIC had an improved resistance profile compared to DTG, EVG, and RAL (with mean fold changes of 2.8, 5.8, >106, and >100, respectively; *P* = 0.04 for BIC versus DTG) and, most notably, against highly INSTI-resistant isolates encoding multiple mutation combinations, such as E92Q/N155H or G140C/S plus Q148R/H/K with or without additional INSTI mutations (*P* = 0.037 for the 23 isolates with mutations at positions 140 and 148 and *P* = 0.031 for the 2 isolates with mutations at positions 92 and 155 for BIC versus DTG) (Fig. 1; see also Table S5). Moreover, BIC displayed ≥2-fold less resistance than DTG against 13 of 47 patient-derived HIV-1 isolates with high-level INSTI resistance, and the remaining 34 isolates had similar fold change values. BIC also retained full susceptibility against 18 HIV-1 clonal variants containing IN sequences with INSTI resistance mutations isolated from 12 subjects with virologic failures in EVG/cobicistat/FTC/TDF (Stribild; Gilead Sciences) clinical trials (see Table S6).

### BIC is active against other drug-resistant mutants of HIV-1.

The antiviral activity of BIC was also determined against a panel of known HIV-1 mutants conferring resistance to NRTIs, NNRTIs, and/or PIs (see Table S7 in the supplemental material). BIC displayed full activity against all tested NRTI-, NNRTI-, and PI-resistant mutants. These results indicate that BIC can be used to effectively treat HIV-1 infections with virus variants resistant to other classes of antiretrovirals.

### BIC selected for HIV variants with low-level resistance.

**FIG 2:**
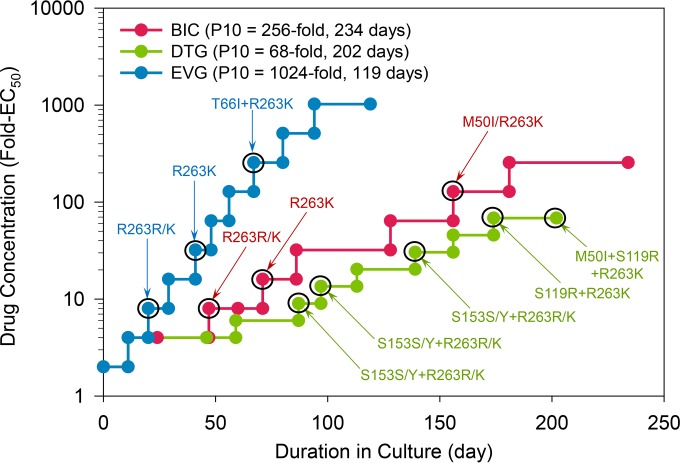
Progress of BIC, DTG, and EVG resistance selection with HIV-1 IIIb. A horizontal line connecting two closed circles represents one passage of the infected cell culture. A vertical line connecting two closed circles represents a transfer of cell-free virus supernatant to fresh uninfected cell culture with either a 1.5-fold or 2-fold increase of the drug concentration. The culture supernatant from the last infected cell culture (black circle) was used for population sequencing, and the mutations identified in the integrase region are indicated. In addition, the viral pool from the last passage of each drug selection was used for clonal sequencing to determine the percentage of clones containing each identified mutation.

The *in vitro* selection of resistance to BIC was performed using two independent approaches based on dose-escalation and breakthrough methodologies. The dose-escalation selection for BIC was conducted in parallel with DTG and EVG under the same conditions. The progress of virus outgrowth in the presence of stepwise increasing concentrations of inhibitors was monitored over time (Fig. 2). The BIC and DTG resistance selections progressed at a rate that was considerably slower than that of EVG, suggesting that BIC and DTG have a higher barrier to resistance emergence than EVG (Fig. 2). The IN coding sequences have been analyzed in selected HIV-1 variants isolated at various stages of the resistance selection process (Fig. 2; see also Table S8 in the supplemental material). In selections with BIC, mutations R263K and M50I successively emerged at passages 3 (P3; day 47) and 8 (P8; day 156), respectively (Fig. 2; see also Table S8). By P9 day 181, 100% of the viruses contained the M50I/R263K double mutant by clonal sequencing. Both R263K and M50I variants are low-frequency natural polymorphisms of HIV-1 IN (43). R263K was first reported to be enriched during *in vitro* selection with EVG (44, 45). It was also selected *in vitro* using DTG and reported to emerge in DTG-treated patients (42), and it confers low-level DTG resistance while decreasing viral fitness (41). The R263K prevalence in treatment-naive HIV-infected patients is extremely low, reaching only ∼0.4% of the analyzed patient population (43). M50I has emerged after R263K in previous *in vitro* DTG selections (41) but has not been observed in patients treated with a first-line regimen containing DTG. In *in vitro* studies, M50I did not restore the loss in HIV-1 infectivity and viral fitness associated with R263K and conferred only moderate resistance to DTG in combination with R263K (46).

During the selection with DTG, mutations S153Y and R263K emerged simultaneously at P4 day 87, followed by S119R and M50I (Fig. 2; see also Table S8 in the supplemental material). By P9 day 174, S153Y waned as S119R concomitantly emerged. At the subsequent final P10 day 202, M50I was added to R263K and S119R. In clonal sequence analyses, 100% of the clones at P10 contained the S119R/R263K double mutant and 65% of the clones contained the M50I/S119R/R263K triple mutant. While S153Y has been observed to coemerge with R263K in DTG selection (41), S119R has not been previously observed in DTG selection. S119R is a natural IN polymorphism prevalent in 1.2% to 6.2% of HIV-1 subtype B isolates from INSTI naive patients (43) (47). It has emerged in HIV patients on INSTI therapy (48) and is associated with coemergence of the T97A mutation, conferring reduced susceptibility to EVG and RAL but not DTG (49). S119R is also associated with other primary INSTI resistance mutations and enhances resistance to INSTIs (47). Residue S119 alteration with Asp was reported to ablate IN strand transfer activity (50), while alterations with Ala or Thr altered the base preference at the site of integration on the target DNA (51).

The EVG selection experiment resulted in the successive emergence of R263K and T66I in P2 day 20 and P6 day 56, respectively. Both of these mutations were previously observed to emerge during *in vitro* selection with EVG (45).

The fold change in the EC_50_ of resistant variants relative to wild-type virus was monitored for several viral passages (see Table S9 in the supplemental material). The viruses sequentially selected with BIC from P3 to P10 displayed small incremental increases in resistance (see Table S9). The P10 isolate (6.1-fold) showed a similar low-to-intermediate cross-resistance to DTG and RAL and a somewhat higher level of resistance to EVG (i.e., ∼16-fold). As expected, none of the viruses selected by BIC showed any cross-resistance to EFV. Similar to BIC, the viruses from P3 to P10 selected with DTG also displayed small incremental increases in resistance to DTG (see Table S9). This P10 isolate (7.9-fold) showed an intermediate level of cross-resistance to BIC and RAL and somewhat higher level of resistance to EVG (i.e., ∼33-fold). Compared to the BIC P10 isolate, the DTG P10 isolate displayed somewhat higher EC_50_ fold-shift values with all tested INSTIs and also encoded the additional S119R substitution. In contrast, the viruses selected with EVG from P2 to P10 displayed a greater incremental increase in resistance in less time, with a 116-fold increase in EVG EC_50_ at P10 (see Table S9). Importantly, although P7, P9, and P10 of EVG-selected virus showed a high degree of resistance to EVG (95- to 116-fold), these viruses conferred only low-level cross-resistance to BIC and DTG (1.3- to 4.0-fold).

The contribution of R263K and M50I to the phenotypic resistance to BIC and of R263K, S153Y, S119R, and M50I to the phenotypic resistance to DTG was assessed with recombinant site-directed HIV-1 mutant variants and is summarized in Table 7. Relative to the wild type, the R263K substitution alone mildly altered the susceptibility to BIC, DTG, and EVG but had no effect against RAL. This result is consistent with our previous resistance profiling results for BIC (Table 6). M50I alone confers virtually no resistance to BIC or the other INSTIs tested. Virus with the M50I/R263K double mutant was equally or slightly less susceptible than virus with the R263K mutant to BIC, DTG, and EVG but remained susceptible to RAL. S153Y substitutions alone conferred resistance similar to that of R263K. The S153Y/R263K double mutant had only slightly increased BIC, DTG, and RAL resistance relative to R263K alone but increased resistance to EVG at 29.5-fold. Clonal sequencing of this P5 virus revealed that even though both S153Y and R263K were detected by population sequencing, no clone actually linked the two mutants. This is consistent with the DTG P5 virus showing only a 4.4-fold resistance to EVG (see Table S9). In contrast, both the S119R/R263K double mutant and the M50I/S119R/R263K triple mutant were prevalent as individual clones in DTG P10 virus. These two mutants displayed slightly elevated BIC, DTG, and RAL resistance relative to that of the R263K mutant and >10-fold resistance to EVG.

**TABLE 7:** Phenotypic profile of resistance mutations selected by bictegravir and DTG

Compound	EC_50_, nM (fold change),^*a*^ by drug and virus variant								
	INSTI selection (WT^*b*^)	BIC/DTG		DTG		BIC	DTG		
		M50I^*c*^	R263K^*c*^	S119R^*c*^	S153Y^*c*^	M50I/R263K^*c*^	S119R/R263K^*c*^	S153Y/R263K^*c*^	M50I/S119R/R263K^*c*^
BIC	1.5 ± 0.3	1.9 ± 0.3 (1.3)	3.2 ± 0.7* (2.1)	2.7 ± 0.7* (1.8)	3.9 ± 0.7* (2.6)	4.2 ± 0.9* (2.8)	4.2 ± 1.3* (2.8)	8.3 ± 1.4* (5.5)	6.4 ± 1.6* (4.3)
DTG	1.4 ± 0.1	2.0 ± 0.2 (1.4)	3.6 ± 0.4* (2.6)	2.1 ± 0.7 (1.5)	4.0 ± 1.1* (2.8)	3.6 ± 0.5* (2.6)	3.6 ± 0.8* (2.6)	6.4 ± 0.8* (4.5)	6.2 ± 2.4* (4.4)
EVG	1.1 ± 0.3	2.0 ± 0.5 (1.9)	5.6 ± 1.8* (5.1)	4.4 ± 3.7 (4.0)	6.1 ± 2.3* (5.5)	7.8 ± 3.5* (7.1)	11 ± 6* (9.6)	33 ± 16* (29.5)	16 ± 3* (14.3)
RAL	7.1 ± 1.1	7.4 ± 1.7 (1.0)	7.7 ± 1.2 (1.1)	15 ± 5* (2.2)	15 ± 4* (2.1)	7.7 ± 2.1 (1.1)	12 ± 2* (1.7)	22 ± 2* (3.2)	19 ± 7* (2.6)
EFV	1.2 ± 0.4	1.1 ± 0.4 (1.0)	1.0 ± 0.4 (0.8)	2.1 ± 1.1 (1.8)	2.2 ± 1.1 (1.8)	1.0 ± 0.3 (0.8)	2.1 ± 1.0 (1.7)	2.0 ± 0.7* (1.7)	3.2 ± 1.8* (2.6)

a EC_50_ ± SD and fold change represent the means from at least 3 independent determinations in triplicate. *, *P* < 0.01 versus the WT.

b Wild-type virus was generated from HIV-1 DNA clone HXB2.

c HIV-1 integrase (IN) mutant.

### Lack of *in vitro* viral breakthrough in the presence of clinically relevant concentrations of BIC.

**FIG 3:**
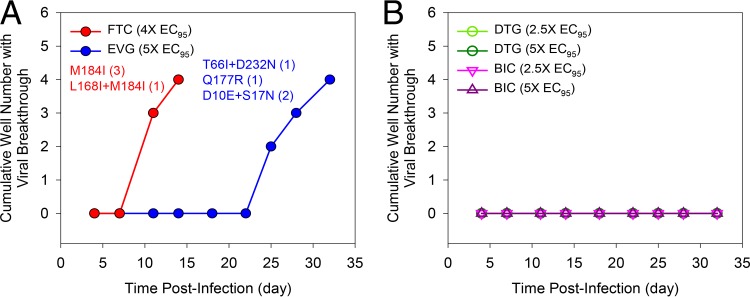
HIV-1 IIIb resistance breakthrough in MT-2 cells. Viral resistance breakthrough for each drug was tested in four independent infected cultures in the presence of constant drug pressure for up to 35 days. The number of cultures with replicating virus based on the observed cytopathic effect was scored at each time point. (A) Viral breakthrough control compounds included emtricitabine (FTC), tested at 4× EC_95_, and elvitegravir (EVG), tested at a *C*_min_ of 5× EC_95_. (B) Dolutegravir (DTG) and bictegravir (BIC) also were tested at 2.5× and 5× EC_95_.

Resistance breakthrough studies using a fixed concentration of drug were conducted with BIC as well as FTC, EVG, and DTG comparator drugs in MT-2 cells (Fig. 3). While HIV-1 broke through FTC (4× EC_95_) and EVG (5× EC_95_) at day 7 and 22 postinfection, respectively, no virus breakthrough was observed at day 32 postinfection with either BIC or DTG when tested at 2.5× EC_95_ and 5× EC_95_. The RT M184I mutation that emerged in FTC breakthrough selection is a known FTC resistance-associated mutation, whereas L168I has not been previously associated with FTC resistance. Of the five mutations that emerged in EVG breakthrough selections, only T66I is a known mutation associated with INSTI resistance. While D10E, S17N, and D232N are HIV IN polymorphic variants not associated with INSTI resistance (43), Q177R is a new variant that has not been previously characterized. The frequency of viral breakthrough in the presence of BIC was also evaluated in primary human CD4^+^ T lymphocytes infected with HIV-1 BaL. In 24 parallel infection samples, BIC suppressed viral breakthrough over a period of 35 days at its cell culture-equivalent clinical minimum (*C*_min_) drug concentration (see Table S10 in the supplemental material), and this effect was similar to observations with DTG and the PI ATV. In contrast, EVG and RAL were associated with a higher incidence of viral breakthrough (54 to 58%) (see Table S10). Virus variants that emerged in the presence of EVG or RAL frequently encoded mutations commonly observed in patients treated with these earlier approved INSTIs (e.g., T66I, E92G/V, Q148R, and N155H). Frequent emergence of drug-resistant HIV variants was similarly observed with both tested NNRTIs (RPV and EFV) and an NRTI (FTC). Viral breakthrough with these agents contained HIV-RT mutations commonly associated with clinical resistance development (e.g., E138K and M230I for RPV, L100I for EFV, and M184I/V for FTC). Collectively, these breakthrough resistance studies indicate that BIC has a barrier to *in vitro* resistance emergence higher than that of EVG or RAL and similar to that of DTG, suggesting that clinical *C*_min_ concentrations of BIC can provide a high barrier to resistance development *in vivo*.

## DISCUSSION

In this report, we describe the biological characterization of BIC, a novel, potent, unboosted inhibitor of the HIV-1 IN strand transfer reaction. BIC inhibits the strand transfer activity of HIV-1 IN enzyme *in vitro* with potency comparable to that of other INSTIs. Accordingly, BIC is an authentic inhibitor of HIV-1 integration in the infected target T cells, as measured by an increased accumulation of abortive 2-LTR circles and a decreased number of virus-host DNA integration junctions in the absence of any effect on the level of late reverse transcription products.

BIC has potent antiretroviral activity in both T-lymphoblastoid cell lines and primary human T-lymphocytes. When tested in primary human PBMCs, BIC displayed consistent antiviral activity across all tested HIV-1 clinical isolates of various subtypes. HIV-2 was similarly susceptible to BIC with an EC_50_ of 1.7 nM. The selectivity (CC_50_/EC_50_) of BIC was high in several cell types. The antiviral activity of BIC in combination with TAF, FTC, or DRV was highly synergistic. Importantly, no antagonistic antiviral interaction was found between BIC and the tested clinically relevant classes of antiretrovirals. These results support clinical investigation of BIC in combination with other antiretroviral agents.

BIC was not active against other viruses and therefore will not promote resistance development for those viruses in HIV-infected patients coinfected with HBV, HCV, or other human viral pathogens. BIC showed low to no cytotoxicity in several non-target human cell lines of different tissue origins, including primary human hepatocytes.

A panel of representative HIV-1 mutant variants with resistance to NRTI, NNRTI, and PI remained fully sensitive to BIC, indicating a good potential for the compound to be clinically active against HIV-1 variants with reduced susceptibility to other antiretroviral classes.

RAL and EVG share similar clinical resistance profiles, with primary mutations often accompanied by compensatory secondary mutations that rescue impaired fitness but further reduce the susceptibility of the mutant viruses to these drugs. In contrast, DTG is characterized by the retention of potency against resistant mutants selected by RAL and EVG (17). We designated nine INSTI-resistant HIV mutants that were used to optimize the resistance profile and screen candidate compounds. These included single mutants at positions E92, Y143, Q148, and N155, representing four principal resistance development pathways against RAL and EVG, and R263K, which is associated with DTG resistance (13, 14, 40), as well as double mutants comprising combinations of mutants at these major positions or with accessory mutations at positions E138 and G140. While Y143R, Q148R, and N155H are primary RAL-associated mutations, E92Q is the most common initial mutation to emerge during failure of EVG-based regimens, followed by N155H and Q148R (52, 53). Our IN mutant screening panel also contained three common double mutants at positions G140/Q148, E92/N155, and E138/Q148, representing 23.6%, 3.2%, and 1.9%, respectively, of clinical specimens from 471 patients with HIV-1 containing RAL-associated mutations (54).

HIV-1 variants resistant to the earlier approved INSTIs remain largely sensitive to BIC (1- to 9-fold change in EC_50_). The Y143R single mutant in our panel has no effect on susceptibility to BIC or DTG (17) and is mildly resistant to EVG but has a higher resistance to RAL (55). BIC maintained activity against all of the single mutants at positions E92, Y143, Q148, N155, and R263, similar to DTG (17, 24, 26). Notably, BIC displayed minimal cross-resistance (1- to 2-fold) to single-mutant HIV-1 variants resistant to RAL and EVG and intermediate cross-resistance (2- to 9-fold) to double-mutant HIV-1 variants highly resistant to RAL and EVG.

Patients who continue on failing regimens with RAL or EVG may accumulate additional mutations that further reduce drug susceptibility, even to DTG. Two DTG clinical studies (VIKING-2 and VIKING-3) in treatment-experienced patients with RAL- or EVG-resistant HIV-1 demonstrated reduced efficacy of DTG dosed twice daily in patients harboring Q148 mutant viruses (24, 26). The greatest reduction in DTG susceptibility is observed with Q148 mutants in conjunction with two or more other mutations (24, 26). Resistance profiling of BIC was assessed with clinical isolates phenotypically resistant to INSTIs, many of which contain mutations conferring high-level resistance to earlier approved INSTIs (>140-fold susceptibility reduction) with a Q148 substitution accompanied by either one or two additional mutations that decrease their susceptibility to DTG by 10- to 63-fold. BIC demonstrated a favorable profile compared to DTG against these highly resistant isolates with a decrease in susceptibility of only 2.6- to 19-fold. Furthermore, BIC had a statistically significant improvement in phenotypic susceptibility compared to DTG in the 25 clinical isolates tested that contained mutations at IN residues 140 and 148.

Parallel *in vitro* dose-escalation and breakthrough resistance selections in the presence of BIC, DTG, and EVG revealed that BIC and DTG have higher barriers to resistance emergence than EVG. In the breakthrough experiments, BIC and DTG did not show viral breakthrough. In the dose-escalation study, the time to resistance development followed the order EVG < BIC < DTG. Furthermore, DTG selected for more resistance substitutions (M50I, S119R, and R263K with a transient S153Y) with higher cross-resistance to EVG and RAL than BIC, which selected for M50I and R263K. Overall, the final selected variants to emerge within the BIC- and DTG-selected cultures had only low-level reduced susceptibility to BIC and DTG (2.8- and 4.4-fold, respectively). EVG selected viruses with high-level resistance (EC_50_ fold changes of up to 139-fold) considerably faster than BIC, and these viruses showed low-level cross-resistance to BIC and DTG (EC_50_ fold changes ranging from 1.3 to 4.0).

M50I and H51Y are two commonly observed secondary mutations associated with the primary mutation R263K during *in vitro* selection with DTG (40). On their own, neither mutation confers resistance to DTG, but in combination with R263K they moderately increase the resistance of R263K (46, 56). In our site-directed mutant analysis, we confirmed that addition of M50I to R263K only showed a modest reduction in susceptibility to BIC over R263K alone while having no effect on susceptibility to DTG. Similar to M50I, S119R on its own confers almost no resistance to DTG or BIC, but in combination with R263K it has an effect comparable to that of M50I. S153Y on its own confers greater resistance to BIC, DTG, and EVG than M50I or S119R but does not appear to be a secondary mutation to R263K, since no clone containing the S153Y/R263K double mutant was observed. Viral clones engineered with the site-directed S153Y/R263K double mutant, although viable, were very difficult to grow, consistent with a reduced fitness relative to variants in drug-selected viral pools.

In conclusion, BIC is a potent and selective novel inhibitor of HIV IN with an improved virology profile relative to all INSTIs currently available in the clinic. These data support its further development as a novel agent in combination with other antiretrovirals for both treatment-naive and treatment-experienced HIV-infected individuals. BIC in combination with FTC and TAF is currently under evaluation in phase 3 clinical trials as a single-tablet regimen.

## Supplementary Material

Supplemental material
