# Supplementary material for: Antiviral Activity of Bictegravir (GS-9883), a Novel Potent HIV-1 Integrase Strand Transfer Inhibitor with an Improved Resistance Profile
Source: Antimicrob Agents Chemother. 2016 Nov 21;60(12):7086–97. doi: 10.1128/AAC.01474-16 (PMC5118987; doi:10.1128/AAC.01474-16)
Supplement: Supplemental material [file supp_60_12_7086__index.html]

Supplemental material 

# Antiviral Activity of Bictegravir (GS-9883), a Novel Potent HIV-1 Integrase Strand Transfer Inhibitor with an Improved Resistance Profile

## Supplemental material

- Supplemental file 1 -

  Supplemental methods and Tables S1 to S10.

  PDF, 545K
