# Supplementary material for: Antiviral Activity of Bictegravir (GS-9883), a Novel Potent HIV-1 Integrase Strand Transfer Inhibitor with an Improved Resistance Profile
Source: Antimicrob Agents Chemother. 2016 Nov 21;60(12):7086–97. doi: 10.1128/AAC.01474-16 (PMC5118987; doi:10.1128/AAC.01474-16)
Supplement: Supplemental material [file AAC.01474-16_zac012165725so1.pdf]

Supplemental Methods

Antiviral Assay Using HIV Clinical Isolates. The antiretroviral activity of BIC was tested against 14 clinical isolates of HIV-1 and one isolate of HIV-2 (from the collection of Southern Research Institute, Frederick, MD) in freshly isolated human peripheral blood mononuclear cells, using the quantitative reverse transcriptase activity endpoint assay. The assay was performed through contract service by the Southern Research Institute (Frederick, MD).

Non-Target Cells Used in Cytotoxicity Assays. The human hepatoma Huh-7 cell line was obtained from ReBLikon GmbH (Mainz, Germany) (Nakabayashi et. al., 1982, Cancer Res. 42:3858-3863). The human hepatoblastoma cell line HepG2, human prostate carcinoma cell line PC-3, and normal fetal lung derived MRC-5 cells were obtained from the American Type Culture Collection (ATCC, Manassas, VA). Huh-7 cells were maintained in Dulbecco's Modified Eagle Medium (DMEM) supplemented with 10% fetal bovine serum (FBS, Hyclone, Logan, UT) and 1% non-essential amino acids (Gibco, Carlsbad, CA). PC-3 and HepG2 cells were adapted to grow in 0.2% galactose-containing, glucose-free Dulbecco's Modified Eagle Medium (DMEM) supplemented with 10% fetal bovine serum (FBS, Hyclone, Logan, UT), 1% non-essential amino acids (Gibco, Carlsbad, CA), 1% Pyruvate (Cellgro), and 1% Glutamax (Invitrogen, Carlsbad, CA). Galactose-adapted cells were maintained in the same culture medium. MRC-5 cells were maintained in Eagle's Minimum Essential Medium (EMEM) supplemented with 10% fetal bovine serum (FBS, Hyclone, Logan, UT). All cell culture media were also supplemented with 100 Units/mL penicillin and 100 μg/mL streptomycin (Gibco).

Human primary hepatocytes were obtained from the following two vendors along with vendor-specific culture medium and supplements: (A) BioreclamationIVT/ Celsis In Vitro Technologies (Catalog # F/M91565, Baltimore, MD); (B) Invitrogen (Catalog # HMFY96, Carlsbad, CA). The cells were seeded at a density of 3.75×10^4 cells per well. Upon arrival, the cells were allowed to recover for 12 – 18 hours in complete medium with added supplement supplied by the vendor at 37ºC in an incubator with 5% CO_2 and 90% humidity before treatment with compounds.

Drug Combination Studies. Compounds were tested in pairwise combinations for their antiviral activity in MT-2 cells. Positive controls, EVG and AZT, as well as negative control (DMSO) were included in every assay plate to define 100% and 0% protection, respectively. The final DMSO concentration in the assay was 0.5%. MT-2 cells were bulk infected with 250-fold diluted HIV-1 IIIb at 100 μL virus per $2x10^6$ cells (i.e. m.o.i. ~ 0.01) and incubated for 3 hours at 37 ºC. Assay plates were incubated for 5-days at 37 ºC in a humidified incubator. The cytopathic effect of HIV was quantified as a luminescence signal after addition of the Cell TiterGlo™ Reagent (catalog # G7571, Promega Biosciences, Inc., Madison, WI). The data were analyzed for the effect of pairwise combination using MacSynergy™ II software (Prichard and Shipman Jr., 1990). The results of the combination studies were expressed as the mean synergy/antagonism volumes (nM^2.%) calculated at the 95% confidence interval from at least three independent experiments in triplicates.

HIV Breakthrough Selection in Human CD4+ T-Cells. $CD4^+$ T-lymphocytes (3×10^6 cells per mL) isolated from the PBMCs of four healthy donors were mixed at 1:1:1:1 ratios and bulk-infected with HIV-1BaL at a concentration of 50 ng p24 equivalent per million $CD4^+$ T cells for 4 hours at 37ºC. After 4 hours of virus adsorption, the cells were washed twice with the RPMI culture medium and seeded into 96-well evaporation control plates (Thermo Fisher Scientific, Waltham, MA) at a cell density of 5×10^5 cells per well in 150 μL RPMI containing 1X IL-2 (5 ng/mL). Plates were incubated at 37°C for 16 hours, after which time a 2X concentration of test compound was prepared in RPMI medium containing IL-2 and 150 μL was added to 24 replicate wells before returning the plates to a humidified 37°C incubator. Drug concentrations were maintained at the tissue culture equivalent of 1X their clinical C_{min} drug concentration determined based on the ratio of free drug fraction in cell culture media and human plasma: BIC (138 nM), DTG (73 nM), EVG (48 nM), RAL (60 nM), RPV (8 nM) and ATV (300 nM). EFV was tested at 1 × and 0.25 × its clinical C_{min} drug concentration (250 nM and 62 nM, respectively), whereas FTC was used at 160 × its EC_{50} obtained in human PBMC, equivalent to 1600 nM. At designated weekly intervals (i.e. 7, 14, 21, and 28 days post-infection), a 200 μL aliquot of cell-free supernatant was harvested from each well and stored at -80°C, followed by replacement with 200 μL of freshly prepared RPMI/drug/IL-2 media supplemented with 3.5×10^5 newly isolated and activated $CD4^+$ T cells. Thirty five days post-infection, the final cell-free supernatants were collected and analyzed for HIV content by using a p24 antigen capture ELISA (Perkin Elmer, Waltham, MA). Viral RNA products were purified from p24-positive supernatants using the QIAamp Viral RNA Mini Kit. The integrase and reverse transcriptase coding regions in each sample were amplified by polymerase chain reaction using the Qiagen OneStep RT-PCR kit and subjected to DNA sequencing (ELIM Biopharmaceuticals, Inc.) to identify any potential resistance-associated mutations.

Anti-HBV Activity and Cytotoxicity Assays in AD38 Cells. AD38 cells cultured in DMEM-F12 were seeded on 96–well Collagen Type I coated plates (Corning BioCoat™, catalog# 356649, Corning, New York, NY) at a density of 5.0×10^4 cells/well. Five days post drug treatment, the supernatant was collected and the cell monolayer was quantified for cytotoxcity with CellTiter-Glo™ Reagent (catalog # G7571, Promega Biosciences, Inc., Madison, WI). The culture supernatant was used for extracellular HBV DNA extraction using the DNeasy 96 Blood and Tissue Kit (catalog# 69582, Qiagen, Valencia, CA). The extracellular HBV DNA was quantified by real–time PCR.

Anti-HCV Activity and Cytotoxicity Assays in HCV Replicon Cell Lines. Compounds are tested for antiviral activity in Huh 7-Lunet derived stable cell lines containing either the HCV1b replicon, hRluc (Con1/SG-hRlucNeo clone 3/3) or the HCV2a replicon, (pRLucNeo2a). These cell lines were maintained in DMEM supplemented with GlutaMAX (catalog# 10566-016, Life Technologies, Grand Island, NY), 10% non-heat-inactivated FBS (catalog# SH3008803HI, Thermo Scientific HyClone, Logan, UT), 1 mg/mL G-418, Pen/Strep, and non-essential amino acids. Compounds were 3-fold serially diluted in 100% DMSO. Following three days of incubation with the test compounds, the activity of Renilla luciferase was quantified using the Dual-Glo luciferase assay system (catalog # E2980, Promega Biosciences, Inc.,

Madison, WI). Compounds were tested for cytotoxicity in the same two replicon cell lines. Three days post incubation with the test compounds, the media was removed and 400 nM Calcein AM (catalog # C3100MP Life Technologies, Grand Island, NY) in PBS was added to each well. Fluorescence resulting from the conversion of non-fluorescent Calcein AM to green fluorescent calcein by live cells was measured in each well.

Anti-HRV Activity and Cytotoxicity Assays in H1-HeLa Cells. For the anti-rhinovirus activity assay, H1-HeLa cells (catalog #CRL-1958, ATCC, Manassas, VA) were trypsinized and resuspended at 50,000 cells/mL in RPMI media supplemented with 10% heat inactivated FBS and 1% Pen/Strep. Viral stocks of HRV1A, HRV14, and HRV16 rescued from pR16.11 (VR-1559, VR-284, and VRMC-8 ATCC, Manassas, VA) were amplified in H1-Hela cells and titrated by $TCID_{90}$. Cells were infected in suspension with a mixture of the three rhinovirus strains at equivalent $TCID_{90}$s and plated onto the 384-well plates (pre-spotted with test compounds) at 1000 cells/well. For the cytotoxicity assay, rhinovirus inoculum was omitted. Plates were incubated in a humidified incubator with 5% CO_2 at 33°C for 3 days and cell viability was measured by CellTiter-Glo™ Reagent (catalog # G7571, Promega Biosciences, Inc., Madison, WI).

Anti-Influenza Virus Activity and Cytotoxicity Assays in NHBE Cells. Normal Human Bronchial /Tracheal Epithelial Cells (NHBE) were grown in BEGM medium supplemented with growth factors (catalog #CC-3170, Lonza, Basel, Switzerland). Test compounds were 3-fold serially diluted in RPMI-BSA medium and 50 µL of the diluted compound were delivered to each well containing 10,000 NHBE cells. Influenza A virus (A/Port Chalmers/1/73, ATCC, Manassas, VA) was added to those cells at 0.117 PFU/cell in 50 µL of RPMI-BSA medium supplemented with 8 µg/mL trypsin (catalog #3750, Worthington, Lakewood, NJ). Five days post incubation, 100 µL of media were removed from each well and replaced with 100 µL of buffer containing 66 mM MES pH 6.5, 8 mM $CaCl_2$, 0.5% NP-40 and 100 µM neuramidase substrate, 2'-(4-Methylumbelliferyl)-α-D-N-acetylneuraminic acid sodium salt hydrate (catalog #M8639-25MG, Gold Biotechnology, St. Louis, MO). One hour post incubation at 37°C, fluorescence was measured using Envision at excitation wavelength of 360 nm and emission wavelength of 450 nm. The cytotoxicity in uninfected NHBE cells is determined in replica plates by adding CellTiter-Glo™ Reagent (catalog # G7571, Promega Biosciences, Inc., Madison, WI) and measuring chemiluminescence.

Anti-RSV Activity and Cytotoxicity Assays in HEp-2 Cells. HEp-2 cells (catalog # CCL-23, ATCC, Manassas, VI) were maintained in MEM media supplemented with 10% FBS and penicillin/streptomycin. For antiviral tests, 100 nL of 3-fold serially diluted compounds were transferred to a 384-well cell culture plate. HEp-2 cells at a density of 50,000 cells/mL were infected by adding RSV strain A2 (catalog # 10-249-000, Advanced Biotechnologies, Columbia, MD) at a titer of $1 \times 10^{4.5}$ tissue culture infectious doses/mL. Immediately following virus addition, 20 µL of the virus/cell mixture was added to the 384-well culture plates and incubated for 4 days at 37°C. Post incubation, the cells were equilibrated at 25°C for 30 minutes. Virus-induced cytopathic effect was determined by adding 20 uL of CellTiter-Glo™ Viability Reagent (catalog # G7571, Promega Biosciences, Inc., Madison, WI) and measuring luminescence using an Envision

plate reader (Perkin Elmer, Waltham, MA). For compound cytotoxicity measurement, the same protocol as for the antiviral activity measurement was used, except that the cells were not infected with RSV.

Clonal Analysis of HIV-1 from Patients. Plasma from 16 patients treated in Studies GS-US-236-0102 and GS-US-236-0103 with confirmed virologic failure and resistance development in the HIV-1 integrase gene were sent to Monogram Biosciences (South San Francisco, CA) for clonal analysis. The HIV-1 RNA was isolated and the cDNA was synthesized using Monogram proprietary primers. Using proprietary primers, PCR amplification of the entire protease, reverse transcriptase, and integrase coding regions of the pol gene were performed. PCR products were inserted into T/A cloning vectors. Approximately 20 clones were chosen per patient sample for sequencing using proprietary primers. A subset of clones was chosen for phenotypic analysis based on the presence of INSTI resistance mutations. Resistance test vectors containing the patient-derived integrase coding region from selected clones were constructed using standard Monogram Biosciences protocols. Phenotypic data for BIC, DTG, EVG, and RAL (EC_{50} values) in these clones were obtained through a contract study by Monogram Biosciences using the PhenoSense integrase assay. The fold-change from the NL4-3 wild-type reference vector values was determined. Phenotypic data was successfully obtained for 18 clones from 12 patients.

Supplemental Tables

TABLE S1. Antiviral Activity of Bictegravir against Hepatitis Viruses

Compound[a]	HCV 1b Replicon Huh-7 cells[a]		HCV 2a Replicon Huh-7 cells[a]		HBV Production AD-38 cells[a]	
	EC_{50} (µM)	CC_{50} (µM)	EC_{50} (µM)	CC_{50} (µM)	EC_{50} (µM)[b]	CC_{50} (µM)
**BIC**	>44	>44	>44	>44	>50	>50
**DTG**	>44	>44	>44	>44	>50	>50
**FTC**	—	—	—	—	0.02	>50
**TFV**	—	—	—	—	0.36	>50
**2-CMeA**	0.112	42.2	0.301	>44	—	—

[a] Mean of 2 independent measurements in triplicate.

[b] Based on the production of extracellular HBV DNA.

TABLE S2. Antiviral Activity of Bictegravir against Respiratory Viruses

Compounds	Influenza NHBE Cells[a]			HRV[c] HI-HeLa Cells[a]		RSV HEp-2 Cells[d]	
	A/PC/1/73[b] EC_{50} (µM)	B/LEE/40[b] EC_{50} (µM)	CC_{50} (µM)	EC_{50} (µM)	CC_{50} (µM)	EC_{50} (µM)	CC_{50} (µM)
**BIC**	53.5	37.2	32.4	>50	>50	>50	31.7
**DTG**	12.8	15.1	8.3	>50	>50	>50	1.6
**2'-FDG**	6.0	14.0	>100	—	—	—	—
**Rupintrivir**	—	—	—	0.03	>10	—	—
**YM-53403**	—	—	—	—	—	0.35	>50

[a] Mean of a single triplicate measurement.
[b] A/PC/173 and B/LEE/40 represent influenza A and B strain respectively.
[c] HRV infection performed with an equal mixture of 3 rhinovirus strains containing HRV1A, HRV14, and HRV16.
[d] Mean of 2 independent measurements in quadruplicate.

TABLE S3. Cytotoxicity of Bictegravir in Non-Target Human Cell Lines and Primary Hepatocytes

Compound	CC_{50} in Cell Lines (µM)[a]				CC_{50} in Primary Human Hepatocytes (µM)[b]	
	Huh7	HepG2	PC3	MRC5	Donor 1	Donor 2
	Human hepatoma	Human hepatoma	Human prostate cancer	Normal human fibroblasts		
**BIC**	43.6	34.6	> 44	> 44	>100	>100
**DTG**	> 44	43.3	> 44	30.7	>100	>100
**Puromycin**	0.72	1.68	0.62	0.36	1.2	0.83

[a] CC_{50} values represent the mean of three independent runs in triplicates.
[b] CC_{50} values were obtained from 2 donors with each assay performed in duplicate.

TABLE S4. In vitro Anti-HIV-1 Activity of Bictegravir in Combination with Selected Antiretrovirals

Drug combination[a]	Synergy/Antagonism[b]		Combination effect
	Type	Mean ± SD	
BIC + BIC	Synergy<br>Antagonism	7.3 ± 10.3<br>-15.1 ± 10.1	Additive
BIC + TAF	Synergy<br>Antagonism	116 ± 27<br>-6.0 ± 5.8	Highly Synergistic
BIC + FTC	Synergy<br>Antagonism	123 ± 56<br>-4.4 ± 6.7	Highly Synergistic
BIC + DRV	Synergy<br>Antagonism	122 ± 41<br>-4.0 ± 10.4	Highly Synergistic
BIC + RAL	Synergy<br>Antagonism	15.6 ± 15.2<br>-10 ± 6.9	Additive
BIC + EVG	Synergy<br>Antagonism	13.0 ± 8.7<br>-6.0 ± 6.1	Additive
DTG + TAF	Synergy<br>Antagonism	119 ± 48<br>-7.6 ± 8.7	Highly Synergistic
DTG + FTC	Synergy<br>Antagonism	130 ± 56<br>-2.0 ± 8.8	Highly Synergistic
DTG + DRV	Synergy<br>Antagonism	108 ± 18<br>-11 ± 11	Highly Synergistic
DTG + RAL	Synergy<br>Antagonism	18.0 ± 15.2<br>-7.0 ± 11.5	Additive
DTG + EVG	Synergy<br>Antagonism	13.0 ± 11.2<br>-4.0 ± 9.7	Additive
EVG + TAF	Synergy<br>Antagonism	120 ± 52<br>-5.7 ± 6.8	Highly Synergistic
RBV + D4T	Synergy<br>Antagonism	9.7 ± 9.5<br>-146 ± 63	Highly Antagonistic

[a] EVG = elvitegravir, RBV = ribavirin, d4T = stavudine, DTG = dolutegravir, TAF = tenofovir alafenamide, FTC = emtricitabine. DRV = darunavir.

TABLE S5. Antiviral Activity of Bictegravir and other INSTIs Against 47 HIV-1 Patient-derived Isolates with INSTI Resistance Mutations.

INSTI Resistance Mutations[a]	Susceptibility (Fold-change vs. WT)[b]				INSTI Resistance Mutations[a]	Susceptibility (Fold-change vs. WT)[b]			
	BIC	DTG	EVG	RAL		BIC	DTG	EVG	RAL
L74M,T97A	0.50	0.64	16	8.48	E92Q,N155H,G163R	2.02	4.12	>150	>143
L68V,Y143C	0.54	0.54	1.9	4.06	G140A,Q148R	2.03	2.22	>150	88
L68L/V,L74M,Y143R	0.59	0.74	26	>143	G140S,Q148H	2.03	3.52	>150	>143
T97A	0.66	0.88	10	1.78	G140S,Q148H	2.12	3.44	>150	>143
T97A,F121Y	0.80	1.63	>150	112	G140S, Q148H	2.17	4.00	>150	>143
T97A,Y143R	0.83	1.11	20	>143	E138K,G140S,Q148H	2.42	3.59	>150	>143
F121Y	0.84	1.05	38	12	G140S,Q148H	2.46	4.73	>150	>143
L74M,N155H	0.90	1.08	103	89	G140S,Q148H, G163K	2.48	5.68	>150	>143
T97A,N155H	0.99	1.51	95	53	G140S,Q148H	2.49	5.56	>150	>143
T97A,Y143C	1.02	1.35	29	>143	E138K,G140S,Q148H	2.52	5.34	>150	>143
E92Q,E157E/Q	1.16	1.41	51	4.8	E138K G140S Q148H	2.62	13	>141	>114
E92Q	1.19	1.58	60	18	G140S,Q148H	2.92	5.46	>150	>143
N155H,E157E/Q	1.23	1.66	28	19	G140S,Q148R	3.01	6.15	>150	>143
E92Q	1.30	1.73	61	6.7	G140S,Q148H	3.81	11	>150	>143
Y143R	1.39	1.50	2.26	22	G140S,Q148H	4.37	13	>150	>143
Y143R	1.39	1.40	2.19	16	T97A,G140S,Q148H	4.39	15	>150	>143
N155H	1.42	2.07	>150	107	E138K,G140C,Q148R	5.32	8.58	>150	>143
Y143C	1.49	1.76	4.24	14	L74L/M,G140A,Q148R	5.38	8.81	>150	>143
T97A,Y143C	1.60	1.47	42	>143	G140S,Q148R	7.05	17	>150	>143
Q148R,E138A	1.69	2.17	>150	43	G140S,Q148H,E138A	7.23	10	>150	>143
N155H,G163R	1.70	1.95	31	15	T97A,G140S,Q148H	7.62	14	>150	>143
E92Q,N155H	1.72	3.49	>150	>143	L74M,G140C,Q148R	8.36	9.06	>150	>143
E138K,Q148R	1.80	2.05	>150	54	E138K,G140A,Q148K	19	63	>150	>143
G140S,Q148H	1.99	3.60	>150	>143					

[a] Primary and other integrase strand transfer inhibitor resistance (INSTI-R) mutations are listed. Primary INSTI-R mutations are T66I/A/K, E92Q/G, T97A, Y143C/H/R, S147G, Q148H/K/R, N155H, and other INSTI-R mutations are H51Y, L68I/V, V72A/N/T, L74M, Q95K/R, F121C/Y, A128T, E138A/K, G140A/C/S, P145S, Q146I/K/L/P/R, V151L/A, S153A/F/Y, E157K/Q, G163K/R, E170A, and R263K in IN.

[b] Susceptibility was determined as the fold-change in EC_{50} vs. NL4-3 wild-type vector by Monogram Biosciences, Inc. The biological or lower clinical cut-offs for reduced susceptibility in this assay are 4.0 for DTG, 1.5 for RAL, and 2.5 for EVG. No cut-off has been determined for BIC. The color shadings subdivide the resistance fold-change into four levels as follows: green, ≤ 2.5-fold; yellow, >2.5-fold; light orange, >5-fold; dark orange, >10-fold.

TABLE S6. Antiviral Activity of INSTIs against 18 HIV-1 Clonal Variants from EVG/COBI/FTC/TDF Treated Patients with Virologic Failure and Emerging NRTI and/or INSTI resistance mutations.

Patient ID-Clone#	INSTI Resistance Mutations[a]	Susceptibility (Fold-change vs. WT)[b]			
		BIC	DTG	RAL	EVG
7332-10	None	0.80	0.87	0.79	0.79
7476-5	T66I, E157Q	0.18	0.25	1.63	22
7476-9	T66I, T97A, E157Q	0.26	0.34	2.45	22
6101-12	Q148R	0.74	0.76	32	123
7425-11	E92Q	0.91	1.29	1.95	37
6101-14	N155H	0.98	1.48	6.56	60
6322-1	N155H, G163R	1.03	1.37	15	35
6648-7	E92Q	1.07	1.46	4.20	43
6648-10	E92Q	1.16	1.60	4.20	42
6041-11	E92Q	1.17	1.53	3.67	26
6667-1	E92Q	1.17	1.21	5.09	57
7562-12	N155H	1.19	1.37	13	35
6322-11	N155H	1.20	1.41	11	42
6101-15	E92Q	1.20	1.26	4.41	57
6648-11	E92Q	1.23	1.57	3.97	32
6503-10	E92Q	1.46	1.87	3.88	25
6545-7	N155H	1.51	1.61	15	41
7299-11	Q148R, G140C	1.52	1.97	24	>208

[a] Primary and other integrase strand transfer inhibitor resistance (INSTI-R) mutations are listed. Primary INSTI-R mutations are: T66I/A/K, E92Q/G, T97A, Y143C/H/R, S147G, Q148H/K/R, N155H, and other INSTI-R mutations are: H51Y, L68I/V, V72A/N/T, L74M, Q95K/R, F121C/Y, A128T, E138A/K, G140A/C/S, P145S, Q146I/K/L/P/R, V151L/A, S153A/F/Y, E157K/Q, G163K/R, E170A, and R263K in IN.

[b] Susceptibility was determined as the fold-change in EC_{50} values vs. NL4-3 wild-type vector by Monogram Biosciences, Inc. The wild-type virus had a mean EC_{50} of 1.55 nM for BIC. The biological or lower clinical cut-offs for reduced susceptibility in this assay are 4.0 for DTG, 1.5 for RAL, and 2.5 for EVG. No cut-off has been determined for BIC. The color shadings subdivide the resistance fold-change into four levels as follows: green, ≤ 2.5-fold; yellow, >2.5-fold; light orange, >5-fold; dark orange, >10-fold.

TABLE S7. Activity of Bictegravir Against NRTI-, NNRTI- and PI-Resistant HIV-1 Mutants

Compound[a]	EC_{50} (nM)[b]	EC_{50} Fold Change Relative to WT[b]											
		NRTI Resistant			NNRTI Resitant					PI Resistant			
	WT	K65R	M184V	6TAMs[c]	K103N	Y181C	Y188L	L100I K103N	K103N Y181C	L10F/ M46I/ I50V	I84V/ L90M	G48V/ I54V/ V82S	G48V/ V82A/ L90M
BIC	1.7 ± 0.5	1.4	1.4	1.4	0.8	1.2	0.9	1	1.2	1.9	1.5	1.1	1.3
DTG	1.6 ± 0.5	1.5	1.6	1.5	0.8	1.4	0.9	1.1	1.4	1.4	1.2	1.0	1.0
EVG	2.5 ± 1.5	1.7	1.5	1.2	1.2	1.2	1.3	1.1	1.1	1.4	1.0	0.9	1.0
FTC	385 ± 225	21.5	>300	10.8	—	—	—	—	—	—	—	—	—
TFV	3,716 ± 790	4.6	1.4	7.1	—	—	—	—	—	—	—	—	—
RPV	0.8 ± 0.4	—	—	—	1.5	5.6	24.5	21.9	9.1	—	—	—	—
EFV	1.2 ± 0.4	—	—	—	45.9	3.6	>83	>83	>83	—	—	—	—
DRV	3.5 ± 1.6	—	—	—	—	—	—	—	—	40	3.1	0.4	0.5
ATV	1.6 ± 0.9	—	—	—	—	—	—	—	—	4.9	114	55	30

[a] RAL = raltegravir, EVG = elvitegravir, DTG = dolutegravir, FTC = emtricitabine, TFV = tenofovir, RPV = rilpivirine, EFV = efavirenz, DRV = darunavir, ATV = atazanavir.

[b] EC_{50} ± SD and EC_{50} fold-changes represent the mean of at least 3 independent determinations in triplicate. The color shadings subdivide the resistance fold-change into four levels as follows: light yellow, >2-fold; bright yellow, >10-fold; light orange, >50-fold; orange, >100-fold.

[c] 6TAMs contains HIV-1 RT mutations M41L, D67N, K70R, L210W, T215Y and K219Q.

TABLE S8. Genotypic Profile of Selected HIV-1 Passages in the Presence of INSTIs

Selected Virus	Mutations[a]	Note[b]
No Drug P8	None	
No Drug P16	None	
BIC P3	R263R/K	Mixture of R and K present at position 263
BIC P5	R263K	R263K is a natural polymorphism associated with INSTI resistance occurring at a frequency of 0.4%. M50I is a natural polymorphism occurring at a frequency of 6.2%
BIC P6	R263K	
BIC P8	R263K, M50I	
BIC P9	R263K, M50I	
BIC P10	R263K, M50I	
DTG P3$_{Lo}$	None	S153Y is a natural integrase polymorphism at a frequency of 0.4% associated with integrase resistance
DTG P4	S153S/Y, R263R/K	Mixture of S and Y present at position 153 and Mixture of R and K present at position 263
DTG P5	S153S/Y, R263R/K	
DTG P7	S153S/Y, R263R/K	
DTG P9	S119R, R263K	S119R is a natural integrase polymorphism at a frequency of 6.2% associated with T97A conferring reduced susceptibility to EVG and RAL but not DTG
DTG P10	M50I, S119R, R263K	M50I is a natural polymorphism at a frequency of 6.2 %
EVG P2	D10D/E, S17S/N, R263R/K	Mixture of (D and E), (S and N) and (R and K) present at positions 10, 17 and 263, respectively.
EVG P4	D10E, S17N, R263K	R263K is a natural polymorphism associated with INSTI resistance occurring at a frequency of 0.4% D10E (89%) and S17N (21%) are natural polymorphisms
EVG P6	D10E, S17N, T66T/I, R263K	Mixture of T and I present at position 66.
EVG P7	D10E, S17N, T66I, R263K	R263K is a natural polymorphism associated with INSTI resistance D10E (89%) and S17N (21%) are natural polymorphisms
EVG P9	D10E, S17N, T66I, R263K	
EVG P10	D10E, S17N, T66I, R263K	

[a] Mutations determined by population sequencing relative to HIV-1 IIIb concensus.

[b] Natural polymorphism frequencies are calculated from Lataillade et. al., 2007.

TABLE S9. Phenotypic Profile of Selected HIV-1 Passages in the Presence of INSTIs

Selected Virus[a]	Duration of Selection	[Drug] Reached[b]	EC_{50} (nM) (Fold-Resistance Relative to HIV-1 IIIb)[c]				
	(day)	(nM)	BIC	DTG	EVG	RAL	EFV
**HIV-1-IIIb**	—	—	2.2 (1)	2.5 (1)	2.1 (1)	8.8 (1)	1.6 (1)
**BIC P3**	47	6	3.8 (1.7)	3.6 (1.4)	5.1 (2.4)	18.1 (2)	1.6 (1)
**BIC P5**	71	12	5.8 (2.6)	6.4 (2.6)	8.3 (3.9)	18.3 (2.1)	1.2 (0.8)
**BIC P6**	86	24	7.8 (3.5)	8.3 (3.4)	7.8 (3.6)	24.4 (2.8)	1.3 (0.8)
**BIC P8**	156	96	11.2 (5.0)	12.0 (4.8)	33.6 (15.8)	28.8 (3.3)	1.7 (1.1)
**BIC P9**	181	192	17.5 (7.9)	19.1 (7.7)	55.0 (25.9)	41.7 (4.7)	2.8 (1.8)
**BIC P10**	234	384	13.5 (6.1)	14.7 (5.9)	33.0 (15.5)	29.3 (3.3)	2.7 (1.7)
**DTG $P3_{Lo}$**	59	6.4	2.9 (1.3)	3.2 (1.3)	1.7 (0.8)	11.8 (1.3)	1.2 (0.8)
**DTG P4**	87	9.6	7.8 (3.5)	7.8 (3.2)	5.0 (2.4)	23.2 (2.6)	1.8 (1.1)
**DTG P5**	97	14.4	10.6 (4.8)	10.3 (4.2)	9.3 (4.4)	28.0 (3.2)	1.7 (1.0)
**DTG P7**	139	32.4	21.8 (9.8)	12.4 (5.0)	15.5 (7.3)	39.7 (4.5)	2.3 (1.4)
**DTG P9**	174	72.9	23.8 (10.7)	15.6 (6.3)	25.1 (11.8)	43.0 (4.9)	2.1 (1.3)
**DTG P10**	202	109.4	30.0 (13.5)	19.7 (7.9)	69.2 (32.5)	56.9 (6.4)	2.8 (1.8)
**EVG P2**	20	7.2	5.8 (2.6)	5.4 (2.2)	17.8 (8.3)	25.9 (2.9)	3.0 (1.8)
**EVG P4**	41	28.8	6.5 (2.9)	7.1 (2.9)	30.2 (14.2)	26.9 (3.0)	2.8 (1.7)
**EVG P6**	56	115	6.8 (3.0)	7.1 (2.8)	48.9 (23.0)	19.9 (2.3)	2.4 (1.5)
**EVG P7**	67	230	8.9 (4.0)	8.1 (3.2)	203.0 (95.4)	28.7 (3.3)	3.6 (2.2)
**EVG P9**	94	922	6.8 (3.1)	6.6 (2.6)	295.9 (139.0)	44.9 (5.1)	3.2 (2.0)
**EVG P10**	119	1843	2.9 (1.3)	3.5 (1.4)	246.4 (115.8)	17.0 (1.9)	2.0 (1.2)

[a] Viral passage numbers are indicated as: P1, P2, P3,…etc.

[b] Resistance selection was initiated at a drug concentration equal to twice the EC_{50} previously determined for each drug (i.e. EC_{50} = 1.5 nM, 1.6 nM and 1.8 nM for BIC, DTG and EVG respectively).

[c] The fold-change is calculated from the ratio of EC_{50} of the selected virus over the EC_{50} of HIV-1 IIIb. The values represent the mean of at least two experiments performed in triplicate.

TABLE S10. HIV-1 BaL Breakthrough Selections in Primary Human $CD4^+$ T-Cells

Drug	Drug Conc. (nM)[a]	p24+ Wells[b]	Frequency of Viral Breakthrough with RAMs	Drug RAMs[c] (No. of incidences)	Other Mutations
BIC (C_{min})	138	0/24	0%	--	--
ATV (C_{min})	300	0/24	0%	--	--
DTG (C_{min})	73	0/24	0%	--	--
EVG (C_{min})	48	23/24	54%	T66I (3), E92V (1), E92G (1), Q148R (1), N155H (3), S230R (1), R263K (3), **no RAMs (10)**	V165I, L172F, A175V, H183R, S283N
RAL (C_{min})	60	12/12	58%	L74M (1), Q148R (2), N155H (2), G163R (1), G163R+F121Y (1), **no RAMs (5)**	V165I, A175T, D253Y
RPV (C_{min})	8	6/24	25%	V90I+E138K (1), K101E (1), V90I+T240I (1), P225L+F227C (1), M230I (2)	R166K, R281K, R281N
EFV (C_{min})	250	2/12	17%	L100I (2)	V118I, R281N
FTC (160 × EC_{50})	1600	14/24	58%	M184I (8), M184V (5), M184T (1)	G112S, E177D, E177K, R281K

[a] Tissue culture equivalent C_{min} = clinical C_{trough} concentration adjusted for human serum protein binding

[b] Viral breakthrough was evaluated in primary $CD4^+$ T-cells after 35 days in culture by p24 ELISA and sequence analysis of p24-positive wells.

[c] The resistance associated mutations (RAM) are found in the coding sequence of the protein target for each drug.
